# Prospects for the Use of Marine Sulfated Fucose-Rich Polysaccharides in Treatment and Prevention of COVID-19 and Post-COVID-19 Syndrome

**DOI:** 10.1134/S1068162022060152

**Published:** 2022-10-29

**Authors:** M. V. Kiselevskiy, N. Yu. Anisimova, M. I. Bilan, A. I. Usov, N. E. Ustyuzhanina, A. A. Petkevich, I. Zh. Shubina, G. E. Morozevich, N. E. Nifantiev

**Affiliations:** 1 Blokhin National Medical Research Center of Oncology, 115552 Moscow, Russia; 2 Laboratory of Glycoconjugate Chemistry, Zelinsky Institute of Organic Chemistry, Russian Academy of Sciences, 119991 Moscow, Russia; 3 Orekhovich Institute of Biomedical Chemistry, 119121 Moscow, Russia

**Keywords:** fucoidan, fucosylated chondroitin sulfate, COVID-19, S-glycoprotein, heparan sulfate

## Abstract

Symptoms of the new coronavirus infection that appeared in 2019 (COVID-19) range from low fever and fatigue to acute pneumonia and multiple organ failure. The clinical picture of COVID-19 is heterogeneous and involves most physiological systems; therefore, drugs with a wide spectrum of mechanism of action are required. The choice of the treatment strategy for post-COVID-19 syndrome is still a challenge to be resolved. Polysaccharides with a high fucose content derived from seaweed and marine animals can form the basis for the subsequent development of promising agents for the treatment of COVID-19 and post-COVID-19 syndrome. This class of biopolymers is characterized by a variety of biological activities, including antiviral, antithrombotic, anticoagulant, hemo-stimulating, anti-inflammatory and immune-regulatory. Low molecular weight derivatives of these polysaccharides, as well as synthetic oligosaccharides with a sufficient amount and sulfation type may be considered as the most promising compounds due to their better bioavailability, which undoubtedly increases their therapeutic potential.

CONTENT

INTRODUCTION

ANTI-CORONAVIRUS ACTIVITY OF SULFATED POLYSACCHARIDES

TREATMENT AND PREVENTION OF IMMUNOSUPPRESSION

ANTICOAGULANT FUNCTIONS OF SULFATED POLYSACCHARIDES

ANTI-FIBROUS ACTIVITY OF SULFATED POLYSACCHARIDES

ANTI-INFLAMMATORY EFFECT OF SULFATED POLYSACCHARIDES

CONCLUSION

## INTRODUCTION

**Fig. 1.**
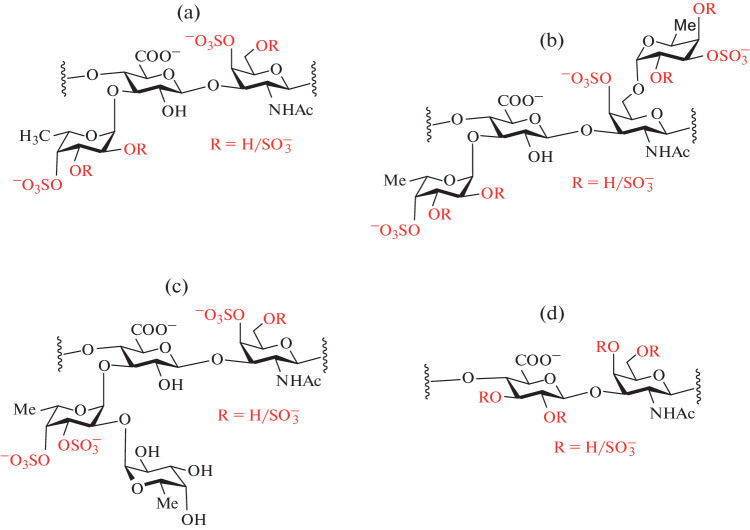
Examples of FCS from holothuria: (a) a polysaccharide from *Cucumaria japonica* [3] that has a trisaccharide repeating unit typical of most known FCS; (b) a fragment of the FCS carbohydrate chain from *Cucumaria frondosa* [4] that has branching both at O-3 of the glucuronic acid and at O-6 of the *N*-acetylgalactosamine residues; (c) a fragment of the FCS carbohydrate chain from *Eupentacta fraudatrix* that has a side chain of (1→2)-linked difucosyl residue [5]; (d) a fragment of another FCS from *Eupentacta fraudatrix* containing 2,3-disulfated glucuronic acid residue [5]. Sulfates in fixed positions and variable substituents R are highlighted in red.

Seaweed and animals contain sulfated polysaccharides of various structures. The most studied polysaccharides with a high fucose content are isolated from echinoderms (sea urchins and sea cucumbers) and from brown algae. Thus, the body wall of holothuria (sea cucumbers) contains two types of these polysaccharides, namely: fucosylated chondroitin sulfates (FCS) and sulfated fucans (SF). FCSs are unique polysaccharides of holothuria. The basis for these molecules is a linear chain constructed from alternating residues of *N*-acetylgalactosamine and glucuronic acid connected in a disaccharide block →3)-β-D-GalNAc-(1→4)-β-D-GlcA-(1→. The structure of this chain does not differ from the structure of the carbohydrate chain of the chondroitin sulfates (CS) of vertebrates [1], however, FCS molecules carry branching, most often in the form of α-L-fucose residues linked to O-3 of glucuronic acid residues. There are also more complex side chains, such as disaccharide blocks consisting of Fuc, Gal and GalNAc residues, bound both to O-3 of glucuronic acid and to O-4 or O-6 of *N*-acetyl-galactosamine. Sulfate groups are usually located at O-4 or O-6 (or simultaneously at O-4 and O-6) of *N*-acetyl-galactosamine residues, have different positions in fucose residues and can sometimes occur at O-2 or O-3 of glucuronic acid residues (Fig. 1) [2–4]. The FCS structures isolated from various holothurian species are specific to these animals [5].

**Fig. 2.**
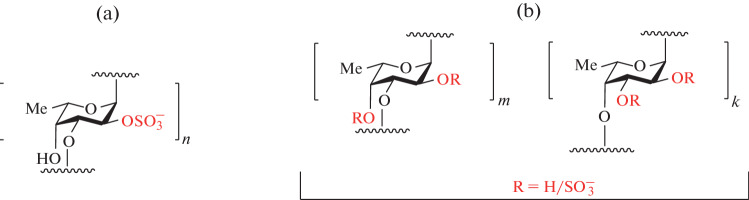
Examples of sulfated fucans from holothuria: a highly regular polysaccharide from *Stichopus horrens* (a) [7] and a mixture of polysaccharides of different structures from *Pattalus mollis* (b) [8]. Sulfates in fixed positions and variable substituents R are highlighted in red.

SF samples were first obtained from sea urchins. They are components of the hydrophilic layer (jelly coat) of the eggs and play a key role in the fertilization process. As a rule, the chains of these polysaccharides are linear and are formed of α-L-fucose residues with different sulfation profile connected via (1→3)-linkages. The specific location of the sulfate groups in the chain usually leads to the formation of a tetrasaccharide repeating units [6]. These polysaccharides can be obtained from sea urchins in very limited quantities, but similar SF together with FCS are found in a more significant amount in the body wall of holothuria. SF from holothuria are more diverse in structure, they are linear chains formed of (1→3)- or (1→4)-linked fucose residues, which may have branchings. Sulfate groups in SF can be located both in the main chain and in the side chains (Fig. 2). Only one type of SF is often found in one species of sea cucumbers [7], though several structurally different SFs may be sometimes isolated as well [8].

**Fig. 3.**
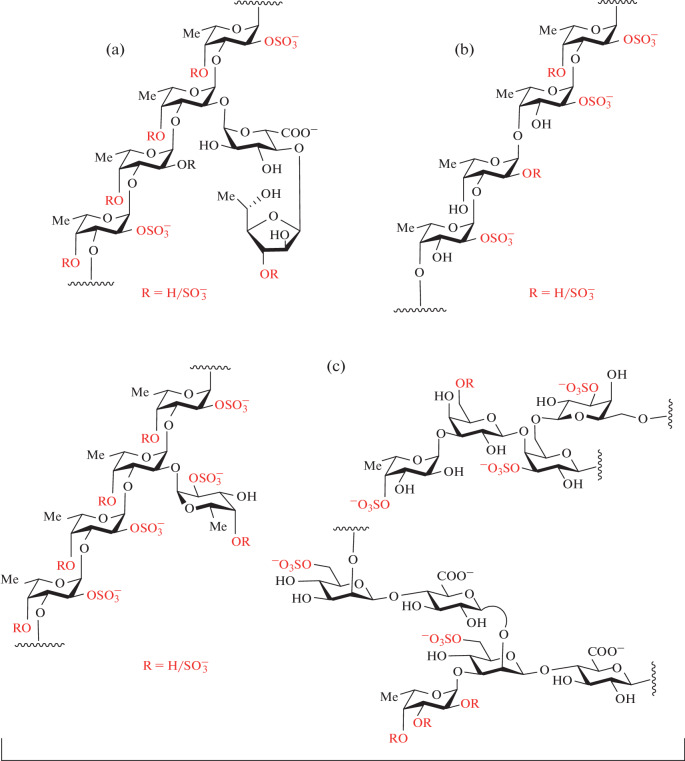
Examples of sulfated polysaccharides produced by brown algae: (a) *Chordaria flagelliformis* [10]; (b) *Fucus evanescens* [11]; (c) *Saccharina latissima* [12]. Sulfates in fixed positions and variable substituents R are highlighted in red.

Brown algae are an almost inexhaustible source of sulfated polysaccharides with a high fucose content, though these biopolymers usually have a more complex structure than FCS and SF. Along with fucose residues, they may contain other monosaccharides, such as galactose, xylose, mannose, glucuronic acid [9]. These heterogeneous polysaccharides were named by the specific term “fucoidans.” The polysaccharide fraction obtained from algae is often a mixture of biopolymers of various structures with SF as the main component. The composition of such a mixture depends on the type and age of the algae, as well as on the growing conditions. Algal SF chains can be formed of repeating (1→3)-linked fucose residues [10] or from alternating (1→3)-and (1→4)-linked fucose chains [11], often with branches in the form of single fucose residues or a variety of short oligosaccharides. Structural regularity is usually masked by an arbitrary arrangement of sulfates or branches. Polysaccharide extract from algae usually contains other sulfated polysaccharides, such as galactofucans, fucoglucuronomannans and fucoglucuronans [12] (Fig. 3).

Sulfated polysaccharides express different biological activities due to their interaction with proteins that determine various physiological processes. The most studied property is an anticoagulant effect similar to that of heparin [13]. Since fucoidans are non-toxic, biocompatible and relatively easily accessible compounds, they are considered as a promising basis for the development of new medicines with antiviral, anti-inflammatory, antitumor, immunomodulatory and anticoagulant activities. Hundreds of articles have already been published regarding the potential therapeutic effect of fucoidans and other polysaccharides with a high fucose content [14–20].

The biological effect of fucoidans is associated primarily with their high degree of sulfation, though other minor features of the structure and molecular weight also play a significant role in their functions. Detailed structural analysis of fucoidans is extremely difficult due to their irregularity and heterogeneity [9]. It is important to note that most of the published biological studies of fucoidans have been performed with commercial samples that are obtained without special purification and confirmation of the chemical structure. As a result, the exact correlation between the structure and biological activity of such products has not been correctly established.

Most of the studies were aimed at the biological activity of sulfated fucose-containing polysaccharides investigating the characteristics of their anticoagulant and antithrombotic effects [21–23]. However, recently, due to the spread of the virus SARS-CoV-2, more attention has been paid to the antiviral functions of these polysaccharides [25–31], which is similar to that of heparin [24]. The review presents a detailed discussion of this problem.

The clinical picture of COVID-19 varies from asymptomatic disease to potentially life-threatening pneumonia, which can eventually lead to acute respiratory distress syndrome (ARDS) [32, 33]. Although the majority of COVID-19 cases can be attributed to mild or moderate disease, about 15% are severe cases requiring oxygen support and about 5% require artificial lung ventilation. Severe Acute Respiratory Syndrome coronavirus 2 (SARS-CoV-2) infects various cells, including alveolar macrophages, which activates them and may induce the cytokine storm [34, 35]. Studies have shown that COVID-19 can significantly affect hematopoiesis and the immune system, leading to lymphopenia, thrombocytopenia, neutrophil dysfunction and anemia [36–39].

Currently, drugs with different mechanisms of action are used for the treatment of COVID-19, which, along with clinical efficacy, can cause undesirable phenomena. A particular problem is to develop an appropriate tactics for the treatment of post-COVID-19 patients requiring long-term supportive care and therapy. Natural medicinal agents are especially interesting since they include biologically active compounds that can be used to develop drugs with a wide range of biological activity with minimal side effects [40]. One of the promising classes of natural compounds are fucoidans with a number of features, such as antioxidant, anti-inflammatory, immune-regulatory, anti-fibrotic, antiviral and anticoagulant activities, as mentioned above, and the potential to stimulate hematopoiesis [41]. Recent studies have demonstrated therapeutic potential of fucoidans in the treatment of COVID-19 and prevention of post-COVID-19 syndrome [17, 42, 43]. A comprehensive review partly described this issue, as well [44]. However, the authors did not consider hemo-stimulating, anti-fibrotic, anticoagulant and anti-inflammatory activities of these polysaccharides, which we analyze in the present review.

## ANTI-CORONAVIRUS ACTIVITY OF SULFATED POLYSACCHARIDES

SARS coronaviruses, despite their diversity, have common functional elements, including spike glycoproteins (SGP, spike glycoprotein, S-protein, S-glycoprotein), typical “spikes” on the surface of the virus resembling a corona, which defined the name of this group of viruses. Previously, interaction of S-protein with angiotensin-converting enzyme 2 (ACE2) was considered as the initial stage of infection [40, 45–47], but recently the researchers have found that the infection starts by the interaction of S-protein with heparan sulfate [24, 48] exposed on the surface of monocytes and macrophages. SARS-CoV-2 can also enter the host cell as a result of S-glycoprotein interaction with another receptor, neuropilin-1 (NRP1) [41, 42, 49, 50].

Specialized laboratories are currently studying the inhibition of SARS-CoV-2 spike glycoprotein interaction with heparan sulfate and ACE2 by sulfated polysaccharides, fucoidans, chondroitin sulfates, fucosylated chondroitin sulfates and others, in order to develop drugs for the COVID-19 prevention and treatment. For instance, in vitro experiments have shown that the fractions of fucoidans RPI-27 (100 kDa) and RPI-28 (~12 kDa) from the seaweed *Saccharina japonica* bind specifically and effectively to the SARS-CoV-2 S-protein and, probably, can prevent binding S-protein to heparan sulfate on host cells and thus suppress viral infection [25]. It is assumed that the antiviral effect of fucoidans is similar to the previously described mechanism of action of heparins [51]. At the same time, polysaccharides from *Saccharina japonica* inhibited SGP SARS-CoV-2 binding more effectively than heparin, and did not have a cytotoxic effect on Vero cells [25].

The studies found that antiviral activity depends on the molecular weight and sulfation degree of polysaccharides. Fucoidans with a higher molecular weight and sulfate content showed more pronounced virus-inhibiting activity. Therefore, molecular weight and degree of sulfation are the two important factors determining the antiviral activity of fucoidans, which was demonstrated by other polysaccharides, as well [26]. Previously, the antiviral activity of fucoidans was shown in relation to other pathogens, such as influenza A virus, hepatitis B virus, canine distemper virus and human immunodeficiency virus [30, 52, 53].

## TREATMENT AND PREVENTION OF IMMUNOSUPPRESSION

Severe COVID-19, like other infections and sepsis, is usually associated with dramatic immune dysregulation characterized by the transition from hyper-inflammatory status to immunosuppression. Similar mechanisms were described for the severe COVID-19 with secondary infection [54]. Severe COVID-19 is associated with the appearance of neutrophil precursors that represent emergency myelopoiesis [55]. This process is characterized by the mobilization of immature myeloid cells, which leads to immunosuppression and, accordingly, to a sharp decrease in the anti-infectious function of the immune system [56]. A close correlation between the disease severity and the lymphopenia severity was observed in COVID-19 patients.

The data showed that lymphopenia is rarely registered in children diagnosed with COVID-19 and with practically zero mortality, whereas in the elderly, where mortality is higher, lymphopenia is more common, especially in severe cases. The studies found that an increased ratio of neutrophils to lymphocytes, the ratio of monocytes to lymphocytes and elevated levels of cytokines such as IL-2R, as well as its ratio to the number of lymphocytes correlated with the severity of the disease and unfavorable prognosis [57]. The patients with severe COVID-19 had depletion of bone marrow progenitor lymphoid cells and accumulation of immature cells. These data indicate that severe COVID-19 leads to the dysregulation of hematopoiesis in the bone marrow [58].

Lymphopenia in patients with severe COVID-19 is associated with a dramatic decrease in natural killer cells (NK) and activated CD8+ T cells, which are the main effectors of anti-infectious immunity [59]. In particular, NK cells have antiviral activity reducing the level of viral protein in the cells infected with SARS-CoV-2 and produce antiviral cytokine IFN-γ.

In addition to antiviral activity, IL-2 activated NK cells confine tissue fibrosis by reducing the expression of pro-fibrotic marker genes COL1A1 and ACTA2 in human lung fibroblasts [60]. Patients with COVID-19 have depleted NK and CD8+ T cells and the recovering patients acquire the restored NK and CD8+ T cell numbers. These facts suggest that functional depletion of cytotoxic lymphocytes is associated with SARS-CoV-2 infection. Therefore, SARS-CoV-2 can disrupt antiviral immunity at an early stage [61, 62]. The researchers suggested that SARS-CoV-2 caused hemolysis and then hypochromic anemia as a result of interaction with the ACE 2, CD147 and CD26 receptors of erythrocytes [63, 64]. Hematopoiesis dysfunction with the decreased number of lymphoid precursors and accumulation of immature and dysfunctional phagocytes in the bone marrow, as well as thrombocytopenia and anemia, requires correction of unbalanced differentiation of bone marrow cells. Sulfated polysaccharides and oligosaccharides may be promising candidates for this role, most of which are safe, as evidenced by acute and chronic toxicity data [65]. A number of studies have demonstrated the immune stimulating effect of oral polysaccharides in adults. For instance, both immunostimulating and suppressing effects, including an increase in stromal factor-1, IFN-g, CD34+ cells and CXCR4-expressing CD34+ cells, as well as a decrease in leukocytes and lymphocytes in the blood, were noted in healthy adults who consumed fucoidans from brown algae *Undaria pinnatifida* (wakame) [66].

Fucoidan from the algae *Chordaria flagelliformis* activates neutrophils for the absorption of bacteria and yeast (the initial phase of phagocytosis), but not the destruction of phagocytized microorganisms mediated by oxygen-dependent mechanisms (completion of phagocytosis).The revealed polysaccharide effect on neutrophils suggests a balanced stimulating effect on cell-mediated anti-infective immunity that excludes excessive release of active oxygen radicals which cause acute tissue damage with organ or multiple organ failure [67]. These conditions stimulated NK cytotoxic activity, as well. The molecular mechanism of the fucoidan stimulating action from *C. flagelliformis* on the effectors of anti-infectious immunity involves integrin CD11c. The ability to induce NK activity, like that of neutrophils, is mediated by stimulating the expression of membrane-bound CB11c molecules on the NKs providing the contact of effectors with target cells.

A number of studies have shown that besides low toxicity, sulfated polysaccharides have the potential to stimulate hematopoiesis. Recent results showed that fucan isolated from the sea cucumber *Holothuria polii* increased the recovery of leukocytes (including neutrophils) in mice after exposure to cyclophosphamide [68]. The authors also noted a tendency to restore red blood cells. Previously, we observed similar effects for fucoidan isolated from the brown alga *C. flagelliformis* [10] and fucosylated chondroitin sulfate isolated from the sea cucumber *Massinium magnum* [69], as well as for their modified derivatives [70, 71]. In addition, the studied polysaccharides could restore the proliferative potential of hematopoietic stem cells of the bone marrow. Taking into account the functions of fucoidans and fucosylated chondroitin sulfates to stimulate hematopoiesis and reduce the manifestations of immunosuppression, as well as to reduce the IL-6 level, it seems appropriate to study these polysaccharides and their derivatives in terms of their potential in complex prevention and immunosuppressive therapy in COVID-19 patients [41].

## ANTICOAGULANT FUNCTIONS OF SULFATED POLYSACCHARIDES

Thrombotic complications are significant causes of unfavorable outcomes of COVID-19 [72]. Hypercoagulation is associated with SARS-CoV-2 direct impact and developing cytokine storm [73]. Patients with COVID-19 have thrombocytosis, an elevated level of D-dimer, a product of fibrin degradation, which correlates with the disease severity and poor prognosis [74]. The data showed that the incidence of thrombotic complications in patients with severe COVID-19 in intensive care units reaches 79% [75, 76]. In addition, a pathomorphological study of patients who died from COVID-19 showed that 58% had undiagnosed deep vein thrombosis, while the immediate cause of death in some cases was a massive pulmonary embolism [77]. Therefore, thrombo-prophylaxis has become an integral part of the therapeutic treatment of COVID-19.

To date, the problem of choosing an anticoagulant, dose and duration of anticoagulant therapy for patients with COVID-19 has not been finally resolved. Moreover, it is not completely clear whether anticoagulant therapy is necessary for outpatient patients and convalescents [78]. However, post-COVID-19 patients often develop coagulopathy in the form of thrombotic events, and these patients require long-term treatment with anticoagulants [79]. Although such drugs reduce the risk of organ and multiple organ failure and death from coronavirus infection, heparin in therapeutic doses did not improve outcomes and had a high probability of side effects in patients with critically poor status [80, 81].

The most significant undesirable effect of anticoagulant therapy is heparin-induced thrombocytopenia (HIT), an immuno–mediated complication leading to transient thrombocytopenia associated with prothrombotic condition [82]. The unfractionated heparin (unfractionated heparin, UFH) or low-molecular-weight heparin (LMWH) is indicated for patients with COVID-19 in hospitals, however, their widespread use may lead to an increase in the frequency of HIT due to hyperstimulation of immunity associated with COVID-19 [83]. An area of uncertainty is the need for prolonged anticoagulant therapy in recovering patients. At this stage of treatment, oral anticoagulants are more preferable than parenteral anticoagulants since that is more comfortable for the patients. At the same time, it is suggested to avoid prolonged anticoagulant therapy to reduce the risk of bleeding [84].

The growing incidence of thrombosis during and after COVID-19 requires further study of the supposed mechanisms and the search for new treatment methods. Numerous studies indicate that sulfated polysaccharides, such as heparin, have anticoagulant and antithrombotic activity that makes this class of compounds as promising drugs for the prevention of thrombosis [85]. Mechanisms of anticoagulant and antithrombotic activity of sulfated fucoidans from seaweed include effects on factors of external and internal coagulation pathways [86]. Despite the similarity of the effects, the mechanism of the antithrombotic action of fucoidans differs from that of heparin and includes an active function at the final stage of coagulation—the conversion of fibrinogen into fibrin determined by the thrombin activity.

At the same time, an important characteristic of low-molecular fractions of fucoidans, which have an antithrombotic effect in venous and arterial thrombosis, is a low hemorrhagic risk [87]. This feature of fucoidans may be important for long-term anticoagulant therapy in COVID-19 and post-COVID-19 patients vulnerable to hemorrhagic reactions. The studies found that low-molecular-weight fucoidans had a noticeable inhibitory effect on thrombin-induced platelet aggregation in vivo and in vitro, while these polysaccharides with an average molecular weight could have a stimulating effect on human platelet aggregation in vitro and had no significant inhibitory effect on thrombin-induced platelet aggregation in rats. These results demonstrated the prospects of low-molecular-weight fucoidans and related synthetic oligosaccharides [88, 89] as potential antithrombotic agents.

## ANTI-FIBROUS ACTIVITY OF SULFATED POLYSACCHARIDES

Pulmonary fibrosis is one of the main complications in patients with COVID-19. Pathogenesis of post-infectious pulmonary fibrosis includes disruption of the pulmonary epithelium and vascular endothelium with uncontrolled fibro-proliferation, as well as a dysregulation of the matrix metalloproteinases release in the inflammatory phase of ARDS. Vascular dysfunction is a key point of development from ARDS to fibrosis, involving vascular endothelial growth factor (VEGF) and cytokines such as IL-6 and TNFα. The epithelium affected by the virus promotes the release of inflammatory mediators via activation of neutrophils and macrophages that control the activation, migration, proliferation and differentiation of fibroblasts, which leads to the production of extracellular matrix components and the destruction of the pulmonary architecture [87]. In addition, the studies reported about trans-differentiation of epithelial cells into fibroblast–like cells defined as epithelial-mesenchymal transition (EMT) [90, 91]. The main EMT inducer is transforming growth factor-β1 (TGF-β1), though various cytokines, chemokines and growth factors are also involved in this process [92]. Despite the fact that at present the anti-fibrotic drugs such as pirfenidone and nantedanib are used in clinical practice, their effectiveness in COVID-19 patients requires further research [93, 94].

A number of studies have shown that fucoidans can prevent the development of pulmonary fibrosis. In particular, it was reported that a fraction of sulfated oligosaccharides MS80 with an average molecular weight of 8 kDa isolated from seaweed inhibited the development of lung fibrosis in rats induced by bleomycin with no signs of toxicity [95]. The use of MS80 led to the decreased pathological parameters and reduced collagen content in the lungs due to competitive inhibition of interaction with TGF-β1 and inhibition of matrix metalloproteinase activity.

The study of a low-molecular fraction of fucoidan from brown algae *Saccharina japonica* showed significant inhibition of bleomycin-induced pulmonary fibrosis in C57BL/6 mice due to a decline of the TGF-β1expression, inhibition of lung EMT, and a decrease of the expression of E-cadherin and fibronectin [96]. Fucoidan isolated from *Sargassum hemiphyllum* inhibited the manifestation of post-radiation pulmonary fibrosis in the irradiated mice (10 Gy/shot) C57BL/6 [91]. The administration of this fucoidan significantly weakened the deposition of collagen 1α, infiltration of lung tissue by neutrophils and macrophages, and also resulted in decreased expression of pro-inflammatory cytokines and chemokines, such as TIMP-1, CXCL1, MCP-1, MIP-2, IL-1RA, TREM-1, SDF-1/CXCL12 and IL-16, in the pleural radiation-induced effusion [97].

It should be noted that pulmonary macrophages express several fibrous mediators and play an important role in the development of fibrosis. They are divided into two phenotypes: classically activated macrophages (M1) with the secretion of Th1 associated cytokines including TNF-α, IL-1β and IL-6 and alternatively activated macrophages M2 with the release of Th2 associated cytokines such as IL-10 and IL-13. Moreover, chemotactic protein 1 of monocytes (MCP-1) determines migration and invasion of macrophages M1 and M2; the chemokine is mainly expressed in alveolar macrophages and participates in cell chemotaxis [98].

Administration of fucoidan isolated from *Sargassum hemiphyllum* to mice with radiation pneumonitis significantly reduced the production of proi-nflammatory cytokines such as TNF-α, IL-1β, and IL-6 by activated macrophages and collagen production by fibroblasts, which correlated with a decrease in infiltration by neutrophils and macrophages of lung tissue. Low-molecular-weight fucoidan isolated from *Laminaria japonica* leveled the manifestations of fibrosis and inflammatory factors in lung tissue in mice stimulated by bleomycin by reducing the expression of β-catenin, TGF-β, TNF-α and IL-6. In addition, this fucoidan blocked the development of the TGF-β1-induced EMT by inhibiting the TGF-β/Smad and PI3K/AKT signaling pathways.

Thus, numerous experimental studies have demonstrated that fucoidans affect all the main chains in the pathogenesis of pulmonary fibrosis, leveling the inflammatory cascade and weakening the process of intercellular matrix formation. These data suggest that fucoidans may be the basis for creating a promising therapeutic agent for the treatment and prevention of post-COVID-19 pulmonary fibrosis [99, 100].

## ANTI-INFLAMMATORY EFFECT OF SULFATED POLYSACCHARIDES

An overreaction of the immune response, a cytokine storm, plays an essential role in the COVID-19 pathogenesis. Besides, overproduction of pro-inflammatory cytokines leads to the development of acute respiratory distress syndrome and multiple organ failure, which are the main cause of death [101, 102]. Favorable outcome of SARS-CoV-2 infection is associated with the administration of steroids, as well as inhibitors or blockers of IL-6 receptors, thus supporting the critical role of the cytokine storm [103]. However, steroids and IL-6 or its receptor inhibitors can cause immunosuppression in patients with COVID-19, and therefore contribute to the suppression of antiviral immunity and lead to secondary bacterial and fungal infections [104, 105]. The risk of death increases in several times in patients with COVID-19 and secondary infection.

Another mechanism of excessive inflammatory response is oxidative stress induced by the production of free radicals by phagocytes in response to viral infection. That, in turn, stimulates macrophages to produce IL-6 cytokine, creating a “vicious circle” [106]. High levels of IL-6 and other inflammatory factors, including C-reactive protein, ferritin, D-dimer and lactate dehydrogenase, are associated with a high risk of death [107]. The published new data showed that severe SARS-CoV-2 infection caused a disruption of the intestinal barrier due to viral infection of the enterocytes and activation of innate and adaptive immunity, contributing to the systemic spread of bacteria and/or microbial products. Translocation of microbial products from the gastrointestinal tract into peripheral blood may increase the hyper-inflammatory status and severity of COVID-19 [108–110] as a result of macrophages activation in the infected tissue and maintaining a cytokine storm during SARS-CoV-2 infection [111]. This assumption is confirmed by the fact that a significant increase of the amount of bacterial lipopolysaccharide (LPS), and soluble sCD14 receptor associated with elevated systemic levels of IL-6, TNF-α, CCL5/RANTES and CCL2/MCP-1 was observed in critically ill patients with COVID-19 [112].

Thus, impaired intestinal barrier function may be one of the mechanisms that contribute to the presence of bacterial toxin and bacterial DNA in the blood of patients with severe COVID-19 [112]. Intestinal bacterial translocation may play an additive/synergistic role in the cytokine release syndrome underlying the adverse development of COVID-19 [113]. Fucoidans have pronounced anti-inflammatory activity [114] and act at various stages of the inflammatory process. One of the possible mechanisms of action of fucoidans is the suppression of the MAPK and NF–kB signaling pathways and the subsequent decrease of the production of pro-inflammatory cytokines and inhibition of selectin activity [115].

Fucoidans also reduce the macrophage determined secretion of pro-inflammatory mediators in response to LPS. Thus, the results showed that fucoidan fractions from *Fucus vesiculosus* inhibited the secretion of TNF-α and IL-1β and neutrophil infiltration that proved their potential to suppress the early stages of inflammation [116]. Another study showed that fucoidan reduced the LPS-induced macrophage production of nitric oxide via mechanism of action similar to that of the anti-inflammatory cytokine IL-10 [117].

Fucoidans can compete with bacterial endotoxin for binding to toll-like receptors and reduce the effect of LPS stimulation [118–120], and also diminish the increased synthesis of PGE2 and pro-inflammatory mediators, such as TNF-α, IL-1β and IL-6 [121]. In particular, fractionated fucoidans from *Ecklonia cava* significantly reduced NO and TNF-α, IL-1β and IL-6 production by LPS-stimulated macrophages [122]. The studies [123, 124] showed that fucoidans from the brown alga *Sargassum horneri* reduced the levels of NO, PGE2 and pro-inflammatory cytokines TNF-α and IL-6 produced by murine macrophages treated with LPS. Other authors [125] reported inhibition of recombinant human cyclooxygenase COX-1 by the fucoidan from *Fucus vesiculosus* *in vitro*.

The fucoidan derived from *Undaria pinnatifida* reduced inflammation in experimental arthritis caused by Freund’s adjuvant in rats [126]. The anti-inflammatory activity of fucoidan from brown algae in vitro may be associated with inhibition of NO synthesis and iNOS expression, decreased secretion of PGE2, TNF-α, IL-1β, IL-6, IL-8. In vivo studies have shown that the anti-inflammatory effect of fucoidan may be associated with a decrease of serum levels of IL-1α, IL-1β, IL-6, IL-10, TNF-α, IFN-γ, PGE2, TGF-β1, myeloperoxidase, a decrease in free radical production, inhibition of neutrophil migration and an increase of IL-10 levels [127]. Fucoidans from *Macrocystis pyrifera* reduced the expression of pro-inflammatory cytokines induced by LPS. Interestingly, the polysaccharide with the lowest molecular weight was the most potent inhibitor of TNF-α and IL-1β secreted by the LPS-stimulated human lymphoid cells. The experiments on mice with experimental colitis showed that orally administered fucoidans can reduce elevated levels of pro-inflammatory cytokines, including TNF-α, IL-1β and IL-6 [128, 129]. Inhibition of the inflammatory cascade suggests a potential therapeutic role of fucoidans as anti-inflammatory drugs for the treatment of COVID-19 patients.

## CONCLUSIONS

Thus, sulfated high- and low-molecular weight polysaccharides with a significant number of fucose chains have a wide range of biological activities such as antiviral, anti-fibrotic, anticoagulant, hemo-stimulating, anti-inflammatory and immune-regulating, which suggests this class of biopolymers as the basis for the drugs aimed at treatment and prevention of COVID-19 and post-COVID-19 syndrome. Low-molecular weight fucoidans and other polysaccharides are the most promising since they demonstrate a better absorption rate and bioavailability that proves their biological potential [30, 130]. Structurally related oligosaccharides of the exact structure can be designed on the base of the information about fucoidan structure [88, 89, 131] and their industrial production is available in compliance with the quality standards of pharmaceutical manufacturing.

The key issue to be resolved is identifying the mechanism of action of the discussed polysaccharides. Obviously, it is associated with their ability to mimic biologically significant sialylated and sulfated oligosaccharide chains on the surface of human cells. It should be noted that the enzymatic processing [132] and chemical synthesis of such compounds, in particular, sialylated ones, is a rather difficult task [132–135]. Thus the sulfated fuco-oligosaccharides which are more accessible for preparative production than the above-mentioned cellular oligosaccharides, can serve as a promising basis in the development of drugs for the treatment and prevention of COVID-19 and post-COVID syndrome.
