# Erratum to: Prospects for the Use of Marine Sulfated Fucose-Rich Polysaccharides in Treatment and Prevention of COVID-19 and Post-COVID-19 Syndrome

**DOI:** 10.1134/S1068162022340015

**Published:** 2022-12-23

**Authors:** M. V. Kiselevskiy, N. Yu. Anisimova, M. I. Bilan, A. I. Usov, N. E. Ustyuzhanina, A. A. Petkevich, I. Zh. Shubina, G. E. Morozevich, N. E. Nifantiev

**Affiliations:** 1 Blokhin National Medical Research Center of Oncology, 115552 Moscow, Russia; 2 Laboratory of Glycoconjugate Chemistry, Zelinsky Institute of Organic Chemistry, Russian Academy of Sciences, 119991 Moscow, Russia; 3 Orekhovich Institute of Biomedical Chemistry, 119121 Moscow, Russia

The article “Prospects for the Use of Marine Sulfated Fucose-Rich Polysaccharides in Treatment and Prevention of COVID-19 and Post-COVID-19 Syndrome”, written by M. V. Kiselevskiy, N. Yu. Anisimova, M. I. Bilan, A. I. Usov, N. E. Ustyuzhanina, A. A. Petkevich, I. Zh. Shubina, G. E. Morozevich, and N. E. Nifantiev, was originally published Online First in Springer-Link on October 29, 2022 without Open Access. After publication, the authors decided to make the article an Open Access publication. Therefore, the copyright of the article has been changed to © The Author(s) 2022 and the article is forthwith distributed under the terms of a Creative Commons Attribution 4.0 International License (http://creativecommons.org/licenses/by/4.0/, CC BY), which permits use, duplication, adaptation, distribution and reproduction of a work in any medium or format, as long as you cite the original author(s) and publication source, provide a link to the Creative Commons license, and indicate if changes were made.

